# Drivers of resting-state fMRI heterogeneity in traumatic brain injury across injury characteristics and imaging methods: a systematic review and semiquantitative analysis

**DOI:** 10.3389/fneur.2024.1487796

**Published:** 2024-11-27

**Authors:** Alexander W. Kashou, Daniel M. Frees, Kaylee Kang, Christian O. Parks, Hunter Harralson, Jesse T. Fischer, Philip E. Rosenbaum, Michael Baham, Christopher Sheridan, Kevin C. Bickart

**Affiliations:** ^1^Department of Radiology, Loma Linda University School of Medicine, Loma Linda, CA, United States; ^2^UCLA Steve Tisch BrainSPORT Program, University of California, Los Angeles, Los Angeles, CA, United States; ^3^Department of Statistics, Stanford University, Stanford, CA, United States; ^4^Department of Kinesiology, Occidental College, Los Angeles, CA, United States; ^5^Department of Neurology, David Geffen School of Medicine at UCLA, Los Angeles, CA, United States; ^6^School of Medicine, University of California, Irvine, Irvine, CA, United States; ^7^Department of Radiology, Wake Forest School of Medicine, Winston-Salem, NC, United States

**Keywords:** resting-state functional MRI, rsfMRI, traumatic brain injury, TBI—traumatic brain injury, neuroimaging, functional connectivity, systematic literature review (SLR)

## Abstract

Traumatic brain injury (TBI) is common and costly. Although neuroimaging modalities such as resting-state functional MRI (rsfMRI) promise to differentiate injured from healthy brains and prognosticate long-term outcomes, the field suffers from heterogeneous findings. To assess whether this heterogeneity stems from variability in the TBI populations studied or the imaging methods used, and to determine whether a consensus exists in this literature, we performed the first systematic review of studies comparing rsfMRI functional connectivity (FC) in patients with TBI to matched controls for seven canonical brain networks across injury severity, age, chronicity, population type, and various imaging methods. Searching PubMed, Web of Science, Google Scholar, and ScienceDirect, 1,105 manuscripts were identified, 50 fulfilling our criteria. Across these manuscripts, 179 comparisons were reported between a total of 1,397 patients with TBI and 1,179 matched controls. Collapsing across injury characteristics, imaging methods, and networks, there were roughly equal significant to null findings and increased to decreased connectivity differences reported. Whereas most factors did not explain these mixed findings, stratifying across severity and chronicity, separately, showed a trend of increased connectivity at higher severities and greater chronicities of TBI. Among methodological factors, studies were more likely to find connectivity differences when scans were longer than 360 s, custom image processing pipelines were used, and when patients kept their eyes open versus closed during scans. We offer guidelines to address this variability, focusing on aspects of study design and rsfMRI acquisition to move the field toward reproducible results with greater potential for clinical translation.

## Introduction

In 2014, the Center for Disease Control and Prevention (CDC) reported 2.53 million traumatic brain injury (TBI) emergency department visits, resulting in 288,000 TBI-related hospitalizations and 56,800 TBI-related deaths (1). TBI is a broad diagnostic category encompassing massive heterogeneity in the injured population as well as injury mechanisms, severities, and chronicities. Despite the prevalence of TBI, this heterogeneity has complicated the search for objective markers of injury and predictors of disability.

Advances in neuroimaging over the last decade have produced powerful tools to detect potential markers of brain injury and recovery. Though standard clinical neuroimaging is useful for identifying structural pathology in TBI, it has limitations in identifying pathophysiological mechanisms of injury-related symptoms and outcomes (2–4). Resting-state functional MRI (rsfMRI) has recently increased in popularity to detect functional alterations in large-scale brain networks (5) that are distinct from structural abnormalities, potentially providing insights into outcomes where other imaging has not. RsfMRI relies on correlated slow wave fluctuations in the blood oxygenation level dependent (BOLD) signal between brain regions as an index of functional connectivity (FC) (6–8). FC between regions has identified several reproducible, large-scale brain networks that map nicely onto groups of brain regions that co-activate in task-based fMRI studies and/or share structural connectivity in diffusion-based MRI in human and non-human primates, such as the default mode, executive, salience, and somatomotor networks (7, 9). Comparisons of FC within networks across healthy and diseased groups can provide insight into functional mechanisms of disease and targets for treatment (5, 10, 11).

In these ways, rsfMRI has the potential to capture diffuse connectional changes to the brain that may be a hallmark of the shearing forces of trauma (12). To date, hundreds of rsfMRI studies have been conducted in TBI, including several reviews on the topic. The prior reviews have been helpful in identifying patterns of connectional differences in specific subgroups of patients with TBI (e.g., chronic pain, headache, severe TBI, blast-related injury), using specific rsfMRI analysis methods, such as graph analysis, or in combination with other imaging modalities, such as diffusion imaging (13–20). Despite the growing popularity of rsfMRI in TBI, the field suffers from heterogeneous findings. Though the prior reviews provide valuable insights into niches within the field, they were not designed to resolve the massive heterogeneity in this literature, which might be achieved through more liberal study inclusion.

We performed a comprehensive systematic review of rsfMRI studies that compared FC between patients with TBI and matched controls. Our primary goal was to perform a broader, more inclusive review of the literature to identify and characterize consistent FC differences that may only become apparent when collapsing across or parsing out the results by injury severity, chronicity, age, population type, as well as network and imaging methods. We selected these factors because they are commonly reported, have face validity, and have been studied as possible confounds or covariates to rsfMRI differences in TBI. For example, studies show that differences in FC occur over time since injury (21, 22), and outcomes vary as a function of injury severity, age, chronicity, and population type (23–27). We attempted to reduce the complexity in this review by organizing results into comparisons of FC within seven of the most commonly studied canonical ICA networks (28).

## Methods

### Identification

**Figure 1:**
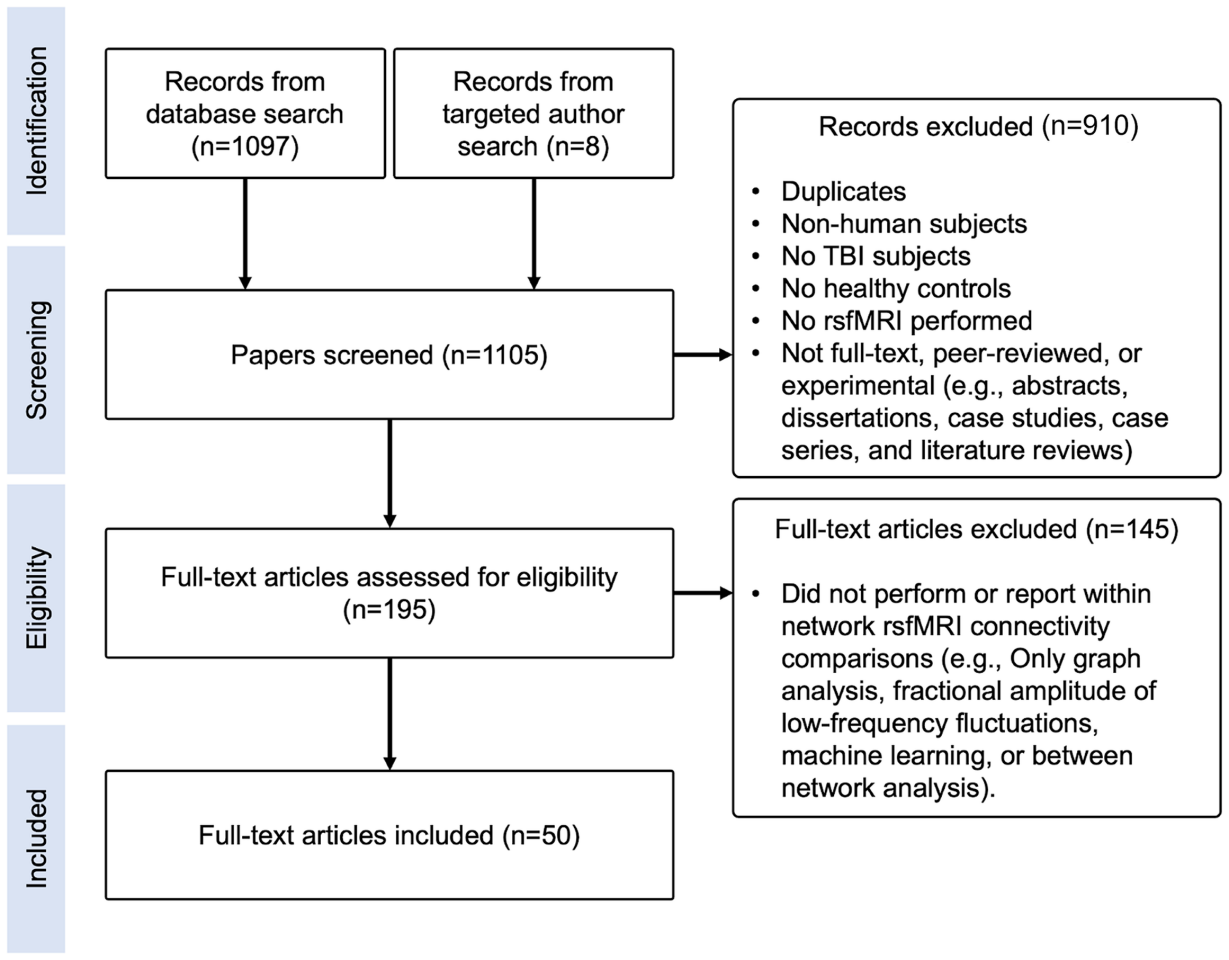
Literature review flow diagram. Diagram depicting identification, screening, eligibility, and inclusion steps performed for this literature review.

This literature review was conducted in accordance with the Preferred Reporting Items for Systematic Reviews and Meta-Analyses (PRISMA) guidelines (29), as depicted in Figure 1. PubMed, Web of Science, Google Scholar, and ScienceDirect were queried with keywords to generate articles. Articles prior to July 2020 were included, yielding 1,097 records (database search in Figure 1). Additional targeted searches were conducted to identify articles from known rsfMRI researchers prior to July 2020 that were not captured by the four database searches, yielding eight records (targeted author search in Figure 1). In total, we identified 1,105 records for screening.

Keywords for PubMed included: (traumatic brain injury [Title/Abstract]) OR (tbi [Title/Abstract]) AND (rsfmri [Title/Abstract]) OR (resting-state [Title/Abstract]). Keywords for Web of Science included: TOPIC: (“resting state fMRI” “traumatic brain injury”) OR (“rsfMRI” “TBI”) OR (“resting state” “MRI” “traumatic brain injury”) OR (“rsfMRI” “traumatic brain injury”) OR (“resting state fMRI” “TBI”) across all years and databases. Keywords for Google Scholar included: “traumatic brain injury” “resting state functional MRI” -rats -dementia -"hepatic encephalopathy” - “task based.” Keywords for ScienceDirect included: Title, abstract, keywords: (“Traumatic brain injury” or “TBI”) AND (“resting state” OR “rs-fmri”).

### Exclusion criteria

Exclusion criteria included: non-human subjects, no TBI subjects, no matched controls, no rsfMRI performed, task-based fMRI only, abstracts, dissertations, case studies, case series, literature reviews, studies that only performed analyses using graph theory, machine learning, other novel methods, or did not report within-network FC results (Figure 1).

### Screening

Query results were converted to the .bib format and imported into a shared folder in Mendeley. Two rounds of selection were performed by evaluating titles and abstracts. Among six reviewers, two independently reviewed each manuscript for inclusion/exclusion criteria. If they disagreed, Dr. Bickart made the final decision. After screening, 910 manuscripts were excluded, 75 of which were duplicates, leaving 195 papers for final eligibility screening (Figure 1).

### Eligibility

Next, the reviewers read each paper completely and excluded an additional 145 papers based on the same inclusion/exclusion criteria as above (Figure 1). In total, three papers via targeted search and 47 papers via database search were included, yielding 50 papers.

### Data extraction

The following information was extracted from each manuscript: mechanism of injury, TBI severity, TBI sample information (age statistics, gender, sample size), control sample information, time post-injury, cross-sectional and/or longitudinal results, scanner manufacturer, tesla strength, coil channel, scan duration, slice thickness, eyes open or closed, sequences acquired during fMRI acquisition, image processing pipeline, software used, rsfMRI metrics computed, analysis type, functional networks/regions investigated, and statistics reported. For longitudinal studies, we extracted the results of cross-sectional, between-group FC comparisons at different time points (i.e., acute, subacute, or chronic), but did not extract within-group FC comparisons over time.

### Defining canonical resting-state networks

**Figure 2:**
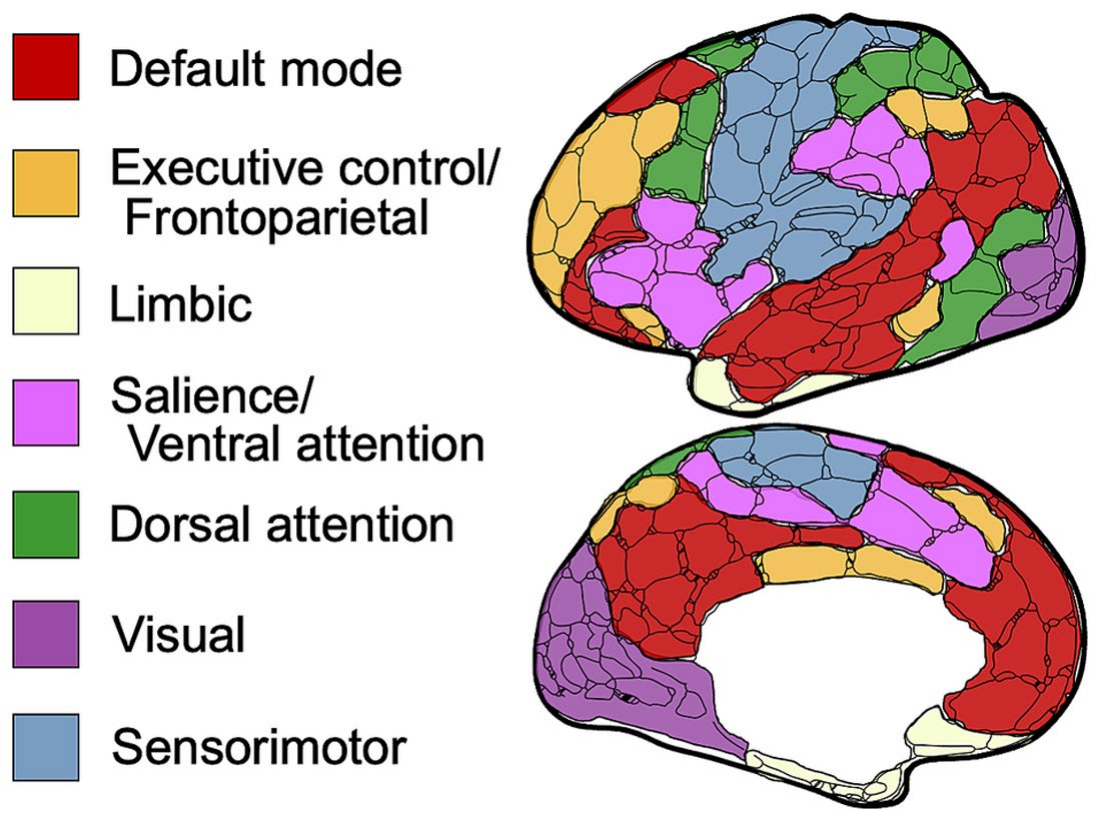
Schematic of resting-state networks used in this review. Visual schematic depicting the anatomical location of each defined canonical brain network.

For comparison purposes, the data extracted from each paper was organized into seven canonical networks based on a widely used and previously defined parcellation scheme (30, 31). The networks included Default Mode (DMN), Executive Control (ECN), Limbic (LN), Dorsal Attention (DAN), Salience (SN) or Ventral Attention (VAN), Visual (VN), and Sensorimotor (SMN) (Figure 2). Findings reported for seed-based or region-of-interest (ROI) analyses were assigned to the network that incorporated that seed or ROI according to the map depicted in Figure 2 and region assignment listed in Table 1. Most studies that outlined their own seed/ROI network assignments adhered to this model. With the data organized into these networks, we could then tabulate results on a comparison-by-comparison basis. Given that studies ranged in the number of networks they analyzed, we report findings for every comparison made across the 50 studies included (Table 2). We only extracted and reported results from the within-network analyses and excluded graph, between-network, and other analyses to enable a semiquantitative summary of results—quantifying the proportion of comparisons per network that were significantly different between TBI and control populations.

**Table 1:** Canonical networks broken down by most commonly referenced brain regions and in accordance with previously defined parcellation schemes (30, 31).

	Network	Brain structures
A	Default Mode Network (DMN)	Medial Prefrontal Cortex (mPFC) Posterior Cingulate Cortex (PCC) Precuneus Angular Gyrus Temporal Poles Temporoparietal Junction (TPJ)
B	Executive Control Network (ECN)	Dorsolateral Prefrontal Cortex (dlPFC) Ventrolateral Prefrontal Cortex (vlPFC) Anterior Cingulate Cortex (ACC)
C	Limbic Network (LN)	Hippocampus Amygdala Pregenual Anterior Cingulate (pgACC) Cingulate Gyrus
D	Salience Network (SN) or Ventral Attention Network (VAN)	Anterior Insula (AI) Dorsal Anterior Cingulate (dACC) Amygdala Ventral Tegmental Area (VTA) or Substantia Nigra Ventral Prefrontal Cortex (vPFC) Ventral Striatum Periaqueductal Gray (PAG) Supramarginal Gyrus
E	Dorsal Attention Network (DAN)	Intraparietal Sulcus (IPS) Superior Parietal Lobule Frontal Eye Fields Ventral Premotor Cortex (vPMC)
F	Visual Network (VN)	Primary Visual Cortex
G	Somatomotor Network (SMN)	Supplementary Motor Area (SMA) Precentral and Postcentral Gyri Paracentral Lobule

In cases where authors did not clearly define the network, these listed structures were utilized to designate results to a network.

**Table 2:** Studies included in this review.

Study citation	Severity	Age	Chronicity	Population type	TBI (*n*)	HC (*n*)
Arenivas et al. (67)	Moderate/severe	Adult	Chronic	Civilian	25	17
Clough et al. (68)	Mild	Adult	Chronic	Sport	15	15
Dailey et al. (69)	Mild	Adult	Chronic	Civilian	15	14
De Simoni et al. (70)	Moderate/severe	Adult	Acute/subacute	Civilian	19	15
Dretcsh et al. (71)	Mild	Adult	Chronic	Military	25	21
Grossner et al. (72)	Moderate/severe	Adult	Subacute/chronic	Civilian	21	23
Guo et al. (73)	Severe	Adult	Acute	Civilian	21	21
Han et al. (74)	Mild	Adult	Chronic	Civilian	40	17
Hou et al. (75)	Mild	Adult	Acute/subacute	Civilian	47	30
Iraji et al. (76)	Mild	Adult	Acute	Civilian	9	15
Lancaster et al. (77)	Moderate/severe	Adult	Chronic	Civilian	21	27
Li et al. (78)	Mild	Adult	Acute	Civilian	58	32
Li et al. (79)	Mild	Adult	Acute	Civilian	50	43
Lu et al. (80)	Mild	Adult	Acute/subacute	Civilian	58	30
Manning et al. (42)	Mild	Adolescent	Subacute/chronic	Sport	14	26
Mayer et al. (44)	Mild	Adult	Acute/subacute	Civilian	26	25
Mayer et al. (81)	Mild	Adult	Subacute	Civilian	48	48
Meier et al. (82)	Mild	Adult	Chronic	Sport	24	44
Messé et al. (83)	Mild	Adult	Subacute/chronic	Civilian	55	34
Militana et al. (84)	Mild	Adult	Acute	Sport	7	11
Murdaugh et al. (46)	Mild	Adolescent	Acute/subacute	Sport	16	12
Nathan et al. (85)	Mild	Adult	Subacute/chronic	Military	15	12
Newsome et al. (86)	Mild	Adolescent	Subacute	Sport	13	13
Newsome et al. (87)	Moderate/severe	Adolescent	Chronic	Civilian	7	9
Newsome et al. (88)	Mixed	Adult	Chronic	Military	17	14
Orr et al. (89)	Mild	Mixed	Chronic	Sport	16	13
Pagulayan et al. (90)	Mild	Adult	Chronic	Military	22	15
Palacios et al. (50)	Moderate/severe	Adult	Chronic	Civilian	20	17
Plourde et al. (91)	Mild	Mixed	Chronic	Civilian	37	20
Rajesh et al. (92)	Mild	Adult	Chronic	Civilian	22	21
Rigon et al. (93)	Mixed	Adult	Chronic	Civilian	21	21
Roy et al. (94)	Moderate/severe	Adult	Subacute/chronic	Civilian	14	12
Santhanam et al. (95)	Mild	Adult	Chronic	Military	51	55
Sharp et al. (96)	Mixed	Adult	Chronic	Civilian	21	23
Shumskaya et al. (97)	Mild	Adult	Acute/subacute	Civilian	35	35
Shumskaya et al. (98)	Moderate/severe	Adult	Chronic	Civilian	43	34
Slobounov et al. (99)	Mild	Adult	Subacute	Sport	17	17
Sours et al. (43)	Mixed	Adult	Subacute/chronic	Civilian	28	28
Sours et al. (100)	Mild	Adult	Subacute/chronic	Civilian	32	31
Stevens et al. (101)	Mild	Mixed	Subacute/chronic	Civilian	27	30
Tang et al. (102)	Mixed	Adult	Not stated	Civilian	12	11
Threlkeld et al. (103)	Severe	Adult	Subacute/chronic	Civilian	17	16
Vakhtin et al. (104)	Mild	Adult	Not stated	Military	13	50
Venkatesan et al. (21)	Moderate/severe	Adult	Chronic	Civilian	22	18
Vergara et al. (105)	Mild	Adult	Subacute	Civilian	47	47
Zhang et al. (106)	Mixed	Adult	Subacute/chronic	Civilian	20	20
Zhou et al. (107)	Mild	Adult	Acute/subacute	Civilian	23	18
van der Horn et al. (108)	Mild	Adult	Subacute	Civilian	54	20
van der Horn et al. (45)	Mild	Adult	Subacute/chronic	Sport	49	20
van der Horn et al. (109)	Mild/Moderate	Adult	Subacute/chronic	Civilian	68	19

### Quality assurance

The original authors of all included papers were emailed to verify our interpretation of the results for this review and to obtain additional statistics for a meta-analysis. Nineteen of the lead authors were able to both verify our interpretation and provide the relevant statistics. This was, however, not enough to perform a meta-analysis.

After the data was validated, the number of reported significant increases, decreases, and no differences in connectivity were manually counted by the reviewers. To ensure accurate transmission of article annotation, code was written to count and organize the data into a database (Literature Review Toolkit: rsfMRI in TBI [Source code]).^1^ The manual counts and code were compared and mistakes were corrected until both were in concordance.

### Parsing the findings into categories

**Figure 3:**
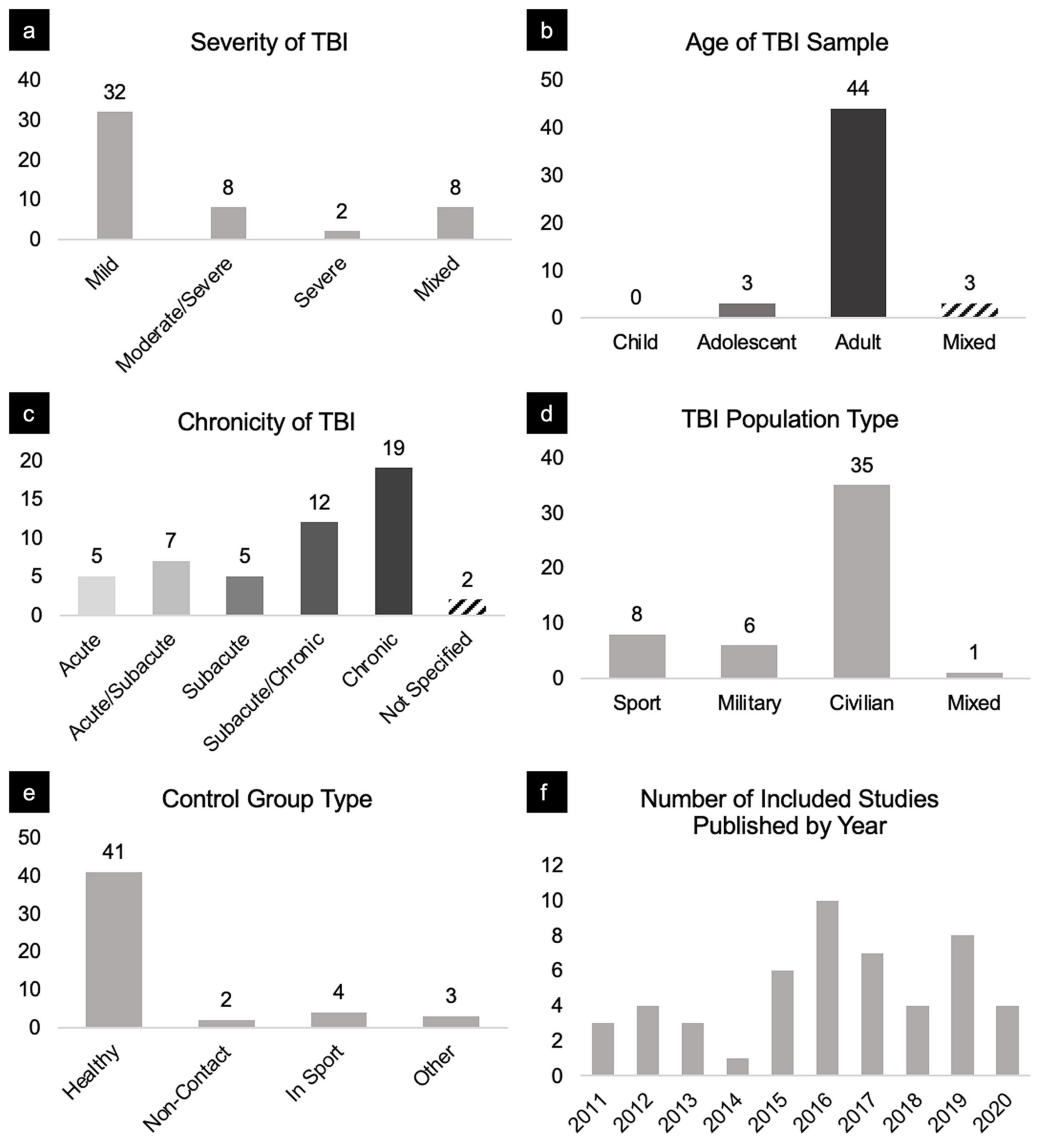
Breakdown of sample characteristics and studies included in this review. Panels portray the distribution of included studies broken down by (a) severity, (b) age, (c) chronicity, (d) TBI population type, (e) control group, and (f) year published.

To determine if findings varied by injury characteristics, demographic variables, or methodological parameters, we parsed the findings into categories: injury severity, age, chronicity, population type, and a variety of imaging parameters (Figure 3). These categorizations are derived from the prevailing consensus across TBI literature.

For severity, we classified results pertaining to TBI patients based on their Glasgow Coma Scale (GCS) scores (32, 33). GCS scores greater than or equal to 13 were classified as mild, GCS scores ranging from 9 and 12 were considered moderate, and GCS scores less than or equal to 8 were considered severe. Some studies included TBI participants of varying severities. Therefore, we also categorized findings as mild/moderate, moderate/severe, or mixed (i.e., mild, moderate, and severe for such cases).

For age, we classified studies involving subjects aged 12–18 as adolescent, age 18 and greater as adult, and otherwise as mixed. Studies that met the inclusion criteria did not identify participants younger than 12 years old.

For chronicity, there is no standard or agreed-upon definition of acute, subacute, and chronic. We chose to classify TBI chronicity based on sources that use biomarkers to track injury progression. For example, such studies (34, 35) define acute TBI as within days, subacute within weeks, and chronic as months to years post-injury. Clinically, there is also some consensus around chronicity as it relates to post-concussion syndrome (PCS). Specifically, PCS refers to a characteristic cluster of symptoms lasting greater than or equal to 3 months after injury (36). We therefore classified scans acquired within the first week post-injury as acute, between 1 and 12 weeks post-injury as subacute, and >12 weeks post-injury injury as chronic. Again, some studies included TBI participants of varying chronicities. Therefore, we also categorized findings as acute/subacute and subacute/chronic to include these comparisons. For longitudinal studies with comparisons between TBI and matched control groups at various times since injury, we treated each comparison as unique, rather than reporting changes in FC over time for either group individually.

For population type, we categorized studies by sport (Sport), military personnel (Military), civilians not injured in sport (Civilian), and mixed if participants were from an array of the aforementioned populations (Mixed).

### Analysis

**Figure 4:**
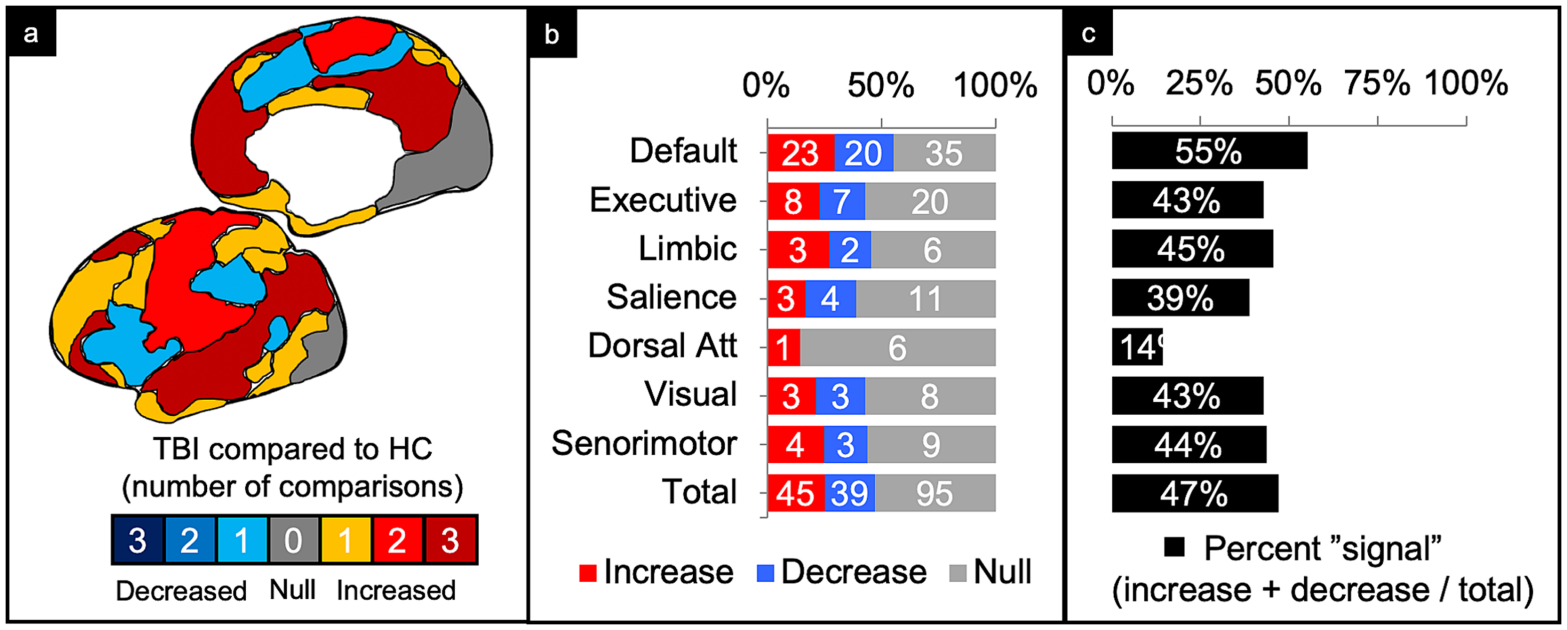
Connectivity comparisons collapsed across injury characteristics. Visual schematic depicting degree of network intensity corresponding to increased or decreased connectivity (a). Comparisons were summed across study and injury characteristics, then stratified by network to display the proportion of increased, decreased, or no (i.e., null) connectivity differences between TBI and control populations (b) and the percent signal of having any significant difference (c).

**Figure 5:**
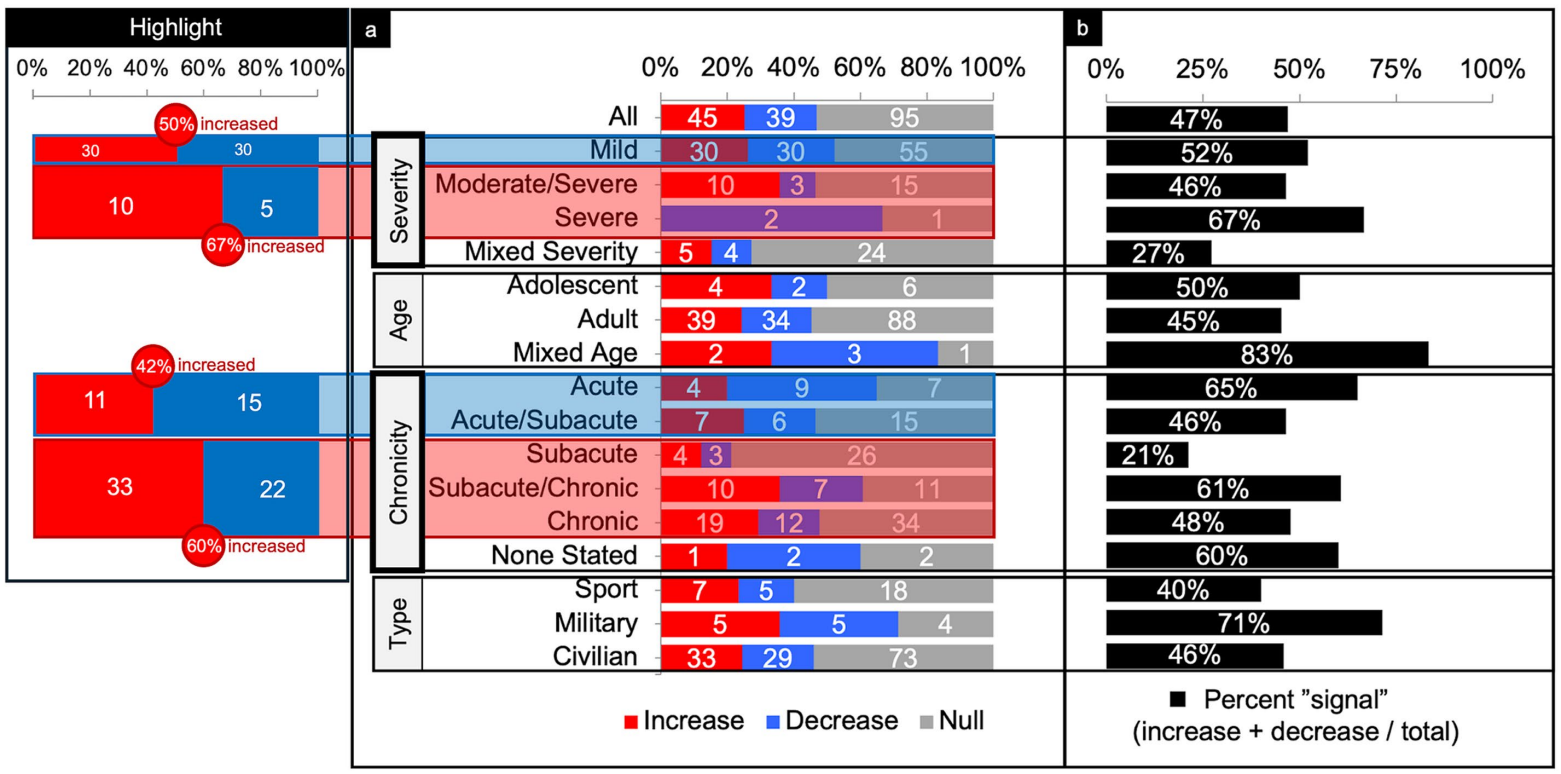
Connectivity comparisons collapsed across networks. Comparisons were summed across study and networks, then stratified by injury characteristics to display the proportion of increased, decreased, or no (i.e., null) connectivity differences between TBI and control populations (a) and the percent signal of having any significant difference (b). The most consistent findings were increased connectivity in the higher severities and chronicities of TBI (Highlight), independent of the other variables.

**Figure 6:**
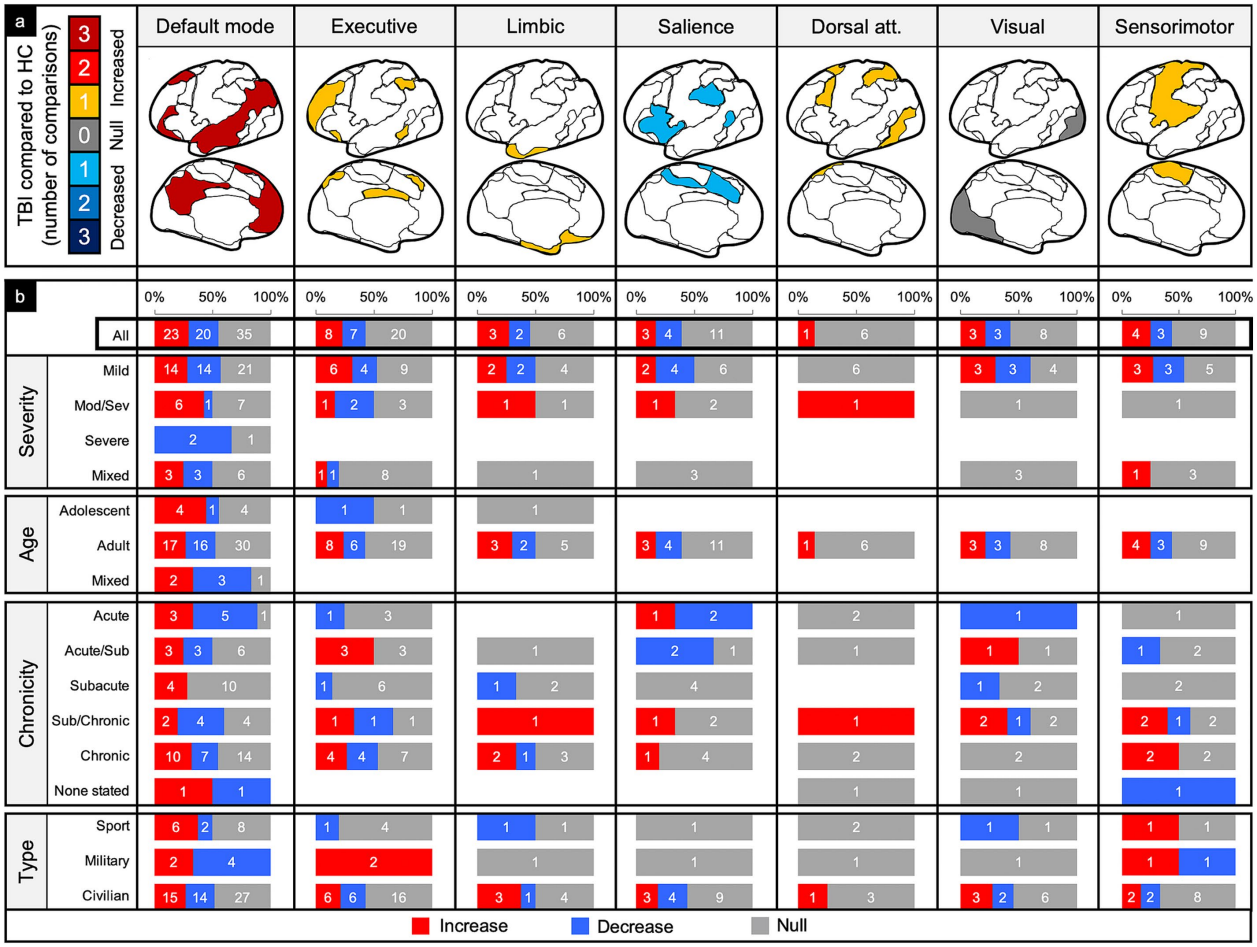
Connectivity comparisons stratified by network and injury characteristics. Comparisons were summed across study, then stratified by networks and injury characteristics to display the proportion of increased, decreased, or no (i.e., null) connectivity differences between TBI and control populations (a) and the percent signal of having any significant difference (b).

We pooled and parsed the findings from comparisons of TBI patients to matched controls, counting the number of reported significant increases and decreases in connectivity or no significant differences in connectivity. Significance was determined by the authors of each study. We would have preferred to perform a meta-analysis to account for differences in effect sizes across studies, but very few studies reported or responded to our inquiries to produce the necessary statistics for this. Comparisons were first collapsed across injury characteristics, such that the connectivity differences could be compared across each of the canonical networks (Figure 4). Next, comparisons were collapsed across networks, allowing for connectivity differences to be compared across injury characteristics (Figure 5). Lastly, connectivity differences were examined across both network and injury characteristics to allow for analysis of each TBI category or demographic within a specified network (Figure 6).

Alongside analysis collapsed by injury characteristics or network, percent global signal was calculated. Percent global signal was defined as the number of increases and decreases in connectivity relative to matched controls divided by the total number of connectivity comparisons. The goal was to use percent signal as a proxy for the degree to which networks reported any difference in connectivity following TBI compared to controls, regardless of whether the connectivity increased or decreased (Figures 4, 5).

## Results

### Studies included

From 1,105 manuscripts, 50 met inclusion criteria, reporting a total of 179 network connectivity comparisons between a total of 1,397 TBI patients and 1,179 matched controls; an average of 3.58 comparisons per manuscript. TBI cohorts primarily included civilian adults with a mild severity of TBI in the subacute to chronic stage (Figure 3).

### Comparisons collapsed across injury characteristics

Of the 179 comparisons, the majority were conducted in DMN (44%) and the least in the DAN (4%) (Figure 4b). Collapsing across injury characteristics and networks, 25% of comparisons showed increased connectivity, 22% showed decreased connectivity, and 53% showed no significant differences between the TBI and control groups, yielding a percent signal of 47% (Figures 4a–c).

### Comparisons collapsed across all networks

#### Severity

Across severity, the majority of comparisons (64%) were performed on patients with mild TBI (Figure 5). Parsing findings by TBI severity showed a trend toward increased connectivity with higher severity. In patients with mild TBI, connectivity differences were evenly split between increased and decreased connectivity (50% increased) with a percent signal of 52%. In higher severities however, more comparisons showed increased than decreased connectivity (60% increased, collapsing across moderate/severe and severe) with roughly comparable percent signal to that of mild TBI (46–67%).

#### Age

Across age, the majority of comparisons (88%) involved adult participants (Figure 5). Percent signal within the adult TBI group was 45%, with a slight tendency toward increased connectivity.

#### Chronicity

Across chronicity, the majority of comparisons (36%) were performed during the chronic phase (Figure 5). Parsing findings by TBI chronicity showed a trend toward increased connectivity with high chronicity. That is, comparisons performed in the acute and acute/subacute phase showed less increased than decreased connectivity (42% increased) whereas comparisons in the subacute through chronic phase showed more increased than decreased connectivity (60% increased). Percent signal across the chronicities was similar (range from 46 to 65%), except for the subacute, only, category (21%).

#### Population type

Across the population type, the majority of comparisons (75%) were performed in civilians (Figure 5). Percent signal across civilians was 46%, with a slight tendency toward increased connectivity (53% increased). Notably, comparisons in military samples yielded a relatively high (71%) chance of finding any difference in FC compared to controls.

### Comparisons within networks

#### Default mode network (DMN)

Of the 78 comparisons between TBI and control groups within the DMN, 55% demonstrated a significant difference in connectivity across all categories of TBI and population factors (Figure 4c), with a slight majority showing increased connectivity (Figure 6, column 1). This pattern of mixed results generally persisted even when parsing the findings by age and population type. Notably, comparisons made in populations with moderate/severe TBI or in the subacute time frame were more likely to show increased connectivity.

#### Executive control network (ECN)

Of the 35 comparisons reported in the ECN, 43% demonstrated a significant difference across all categories of TBI and population factors (Figure 4c), with a nearly even split between increased and decreased connectivity (Figure 6, column 2). This pattern of mixed results persisted when parsing the data by severity, age, and population type. Notably, comparisons made in the acute/subacute time frame revealed solely increased connectivity when a significant difference was detected.

#### Limbic network (LN)

Of the 11 comparisons reported in the LN, 45% demonstrated a significant difference across all categories of TBI and population factors (Figure 3c), with a nearly even split between increased and decreased connectivity (Figure 6, column 3). This pattern of mixed results persisted when parsing the data by severity, age, chronicity, and population type.

#### Salience network (SN)

Of the 18 comparisons reported in the SN, 39% demonstrated a significant difference across all categories of TBI and population factors (Figure 4c), with a nearly even split between increased and decreased connectivity (Figure 6, column 4). This pattern of mixed results persisted when parsing the data by severity, age, chronicity, and population type. Notably, comparisons made in the SN showed the second lowest percent signal of all the networks across all categories of TBI and population factors.

#### Dorsal attention network (DAN)

The DAN was the least studied network. Of the seven comparisons reported in the DAN, 14% demonstrated a significant difference across all categories of TBI and population factors (Figure 4c), showing only increased connectivity (Figure 6, column 5).

#### Visual network (VN)

Of the 14 comparisons reported in the VN, 43% demonstrated a significant difference across all categories of TBI and population factors (Figure 4c), with an even split between increased and decreased connectivity (Figure 6, column 6). This pattern of mixed results persisted when parsing the data by severity, age, chronicity, and population type.

#### Sensorimotor network (SMN)

Of the 16 comparisons reported in the SMN, 44% demonstrated a significant difference across all categories of TBI and population factors (Figure 4c), with a nearly even split between increased and decreased connectivity (Figure 6, column 7). This pattern of mixed results persisted when parsing data by severity, age, chronicity, and population type.

### Comparisons organized by methodology

#### Eye methodology

**Figure 7:**
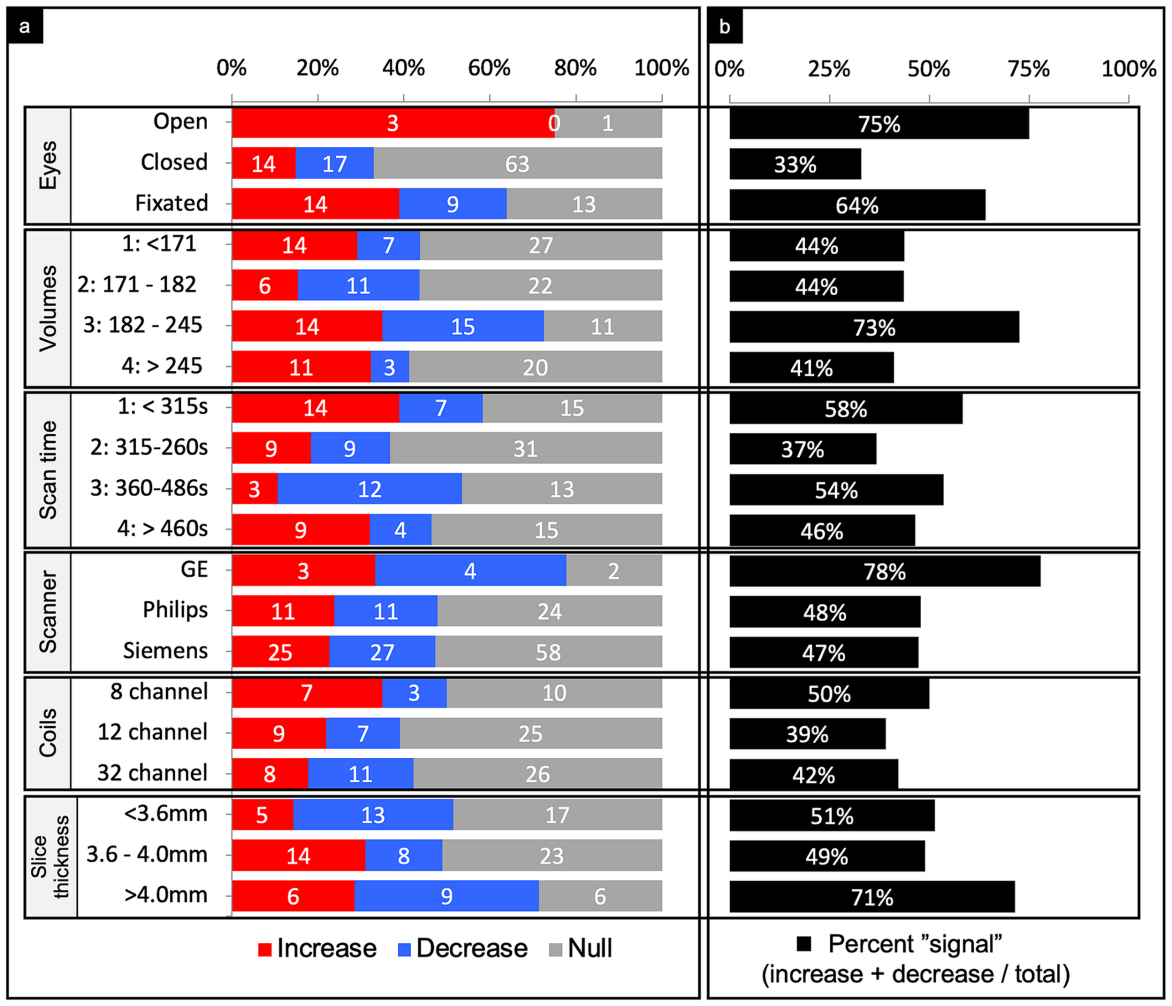
Connectivity comparisons stratified by scanner methodology. Comparisons were summed across study, networks, and injury characteristics, then stratified by imaging methodology to display the proportion of increased, decreased, or no (i.e., null) connectivity differences between TBI and control populations (a) and the percent signal of having any significant difference (b). For the number of volumes and scan time, we stratified the comparisons by quartiles. For the slice thickness, we stratified comparisons by tertiles. All methodological variables listed refer to the rsfMRI sequence only.

The majority of studies (75%) reported whether subjects were instructed to keep their eyes closed, open, or fixated during the scan (Figure 7). Of the comparisons conducted from scans instructing subjects to have their eyes closed (53%), 33% showed a significant difference between TBI and control samples. Of those that found a difference, there was a slight majority of decreased connectivity (55%).

#### Number of volumes

Quartiles were generated based on the distribution of the number of volumes taken. Observations were then sorted according to the quartile range they fell into (Figure 7). 10% of the comparisons did not report the number of volumes acquired during the scan. Among the 3rd quartile of scan volumes, percent signal was 72.5%, which was noticeably higher than the other quartiles. Of those that found a difference in the 3rd quartile, findings of increased or decreased connectivity were roughly equal.

#### Scan time

Quartiles were generated based on the distribution of the scan duration in seconds. Observations were then sorted into the quartile range they fell into (Figure 7). 21% of the comparisons did not report the scan duration. Among the second quartile of scan duration, percent signal was 37%, which was noticeably lower than the other quartiles. Of those that found a difference in the 2nd quartile, findings of increased or decreased connectivity were equal.

#### Scanner manufacturer

Other than three studies (14 comparisons), all used a single scanner for acquisition, which included either GE, Philips, or Siemens (Figure 7). The majority used Siemens. Results were roughly equally mixed across increased, decreased, and null when organized by scanner manufacturer.

#### Coil channels

Only 30 studies (61% of comparisons) reported the number of coil channels used. The majority used the 32 channel coil followed by a 12 channel coil (Figure 7). As above, the findings were roughly evenly mixed results across coil channel number.

#### Slice thickness

Thirty four studies (56% of comparisons) reported the slice thickness. Tertiles were generated based on the distribution of slice thickness in millimeters (Figure 7). Among the 3rd tertile of slice thickness, percent signal was 71%, which was noticeably higher than the other two tertiles.

#### Tesla strength and acquisition sequence

We did not parse findings by tesla strength or acquisition sequence given they did not differ across the studies. Specifically, all 50 studies used a 3-Tesla MRI machine and acquired a T2 gradient-echo, echo-planar (EPI) sequence. No other fMRI sequence was consistently reported.

#### Preprocessing software

**Figure 8:**
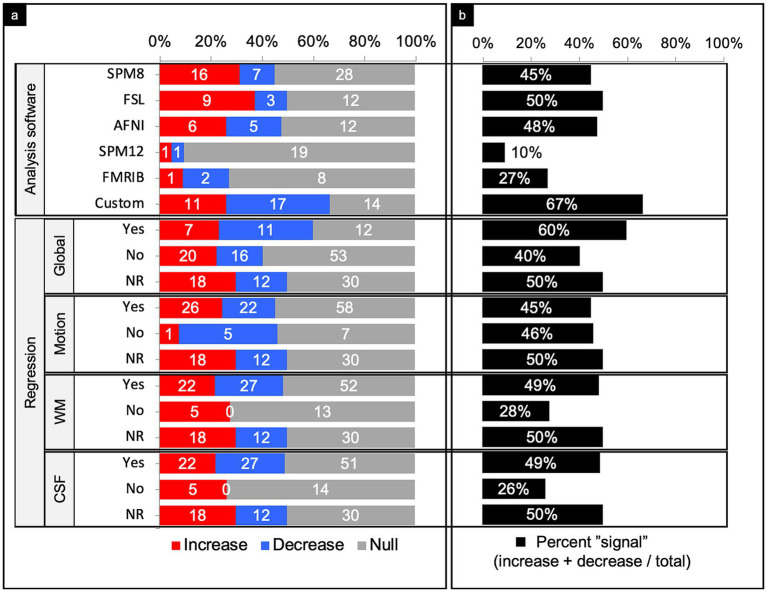
Connectivity comparisons stratified by image analysis. Comparisons were summed across study, networks, and injury characteristics, then stratified by image analysis to display the proportion of increased, decreased, or no (i.e., null) connectivity difference between TBI and control populations and (a) and the percent signal of having any significant difference (b). NR, not reported; WM, white matter; CSF, cerebrospinal fluid.

A variety of preprocessing software was used across the literature. The five most common softwares used were SPM8 (28%), FSL (13%), AFNI (13%), SPM12 (11%), and FMRIB (6%) (Figure 8). Further, 23% of comparisons combined multiple preprocessing softwares or used their own custom pipelines, usually based in MATLAB.

SPM8 yielded a percent signal of 45%, with nearly 70% of comparisons with connectivity differences showing increased connectivity. In contrast, SPM12 yielded a percent signal of just ~10%. FSL and AFNI both yielded a percent signal of around 50%. Of the significant differences in studies that used FSL, 75% detected an increase in connectivity. Of the significant differences in studies that used AFNI, there was a near even split of increased and decreased connectivity. Studies that used mixed methods or custom pipelines yielded the highest percent signal of 67%.

#### Signal regression

Of the 179 comparisons, the authors only reported whether they performed signal regression in 119 comparisons (66%) (Figure 8). Overall, performing a signal regression in most categories, other than motion, revealed an increased sensitivity for finding a significant difference in connectivity between people with TBI and matched controls. Among those that performed white matter and cerebrospinal fluid (CSF) regression, percent signal was notably higher than those who did not perform these regression methods. Of those that did not perform white matter or CSF regression, all comparisons that yielded a signal showed increased connectivity. It should be noted that nearly every study with the exception of 1 utilized white matter and CSF regression in tandem, and thus had roughly equal distributions.

## Discussion

Traumatic Brain Injury (TBI) can cause both focal and diffuse brain changes. Whereas standard clinical neuroimaging identifies structural pathology, it does not reveal functional alterations that may underlie poorer outcomes. Resting-state fMRI has been used to detect functional alterations in network architecture unique to TBI, offering potential insights where other imaging methods have not. However, despite its growing use, there is no clear consensus on functional connectivity (FC) differences between TBI and control populations. To address this gap, we performed a systematic review to identify driving factors that may explain the heterogeneity when comparing FC between TBI and control populations. Specifically, we combined and stratified findings by injury severity, age, chronicity, as well as the studied population type, network, and imaging methods across 50 studies that performed 179 comparisons in 1,397 TBI patients and 1,179 matched controls.

The most consistent findings were a trend toward increased connectivity in chronic stages of TBI and those with more severe head injuries, independent of the other variables (Figure 5). Increased connectivity may reflect compensatory processes that are only needed with more severe or chronic injury states (37–39). We also found a handful of trends when examining individual networks collapsed across all other factors. Otherwise, we found little consensus in the direction or significance of connectivity differences. Based on our findings, we provide recommendations for future work in efforts to standardized rsfMRI imaging in TBI and lead the field toward more reproducible results.

### Collapsing across all variables

When collapsing across all networks, injury characteristics, patient demographics, and imaging techniques (Figure 4b, “Total” row), the findings were generally mixed. There were about an equal number of comparisons showing a significant difference versus no difference in connectivity (47% signal) between patients with TBI and matched controls. Of the significant differences, again, the findings were fairly mixed, but with slight predominance of increased connectivity (45 increased and 39 decreased).

### Severity

Parsing the comparisons by severity revealed that patients with severe and moderate/severe TBI were more likely to show increased (67%) over decreased connectivity (33%) as compared to controls, whereas patients with mild TBI showed a 50/50 split between increased and decreased connectivity as compared to controls (Figure 5). It may be that higher severity injuries disrupt more axonal connections, warranting compensatory processes (37–39), such as cortical reorganization, neurogenesis, axonal sprouting, and angiogenesis, which have been demonstrated in animal models (40, 41).

Surprisingly, however, studies show a similar likelihood of finding any significant difference in FC whether in mild TBI (52%) or moderate/severe TBI combined (56%) as compared to controls (Figure 5). Despite the obvious difference in structural and neurological consequences of injury between these TBI severities, the differences in FC as compared to controls is not as apparent. Studies directly comparing FC in patients across TBI severities are lacking.

### Chronicity

Parsing the comparisons by chronicity revealed a cross-sectional trend of increased connectivity by time since injury. Because we dealt with longitudinal studies as discrete, between-group comparisons of FC at acute, subacute, or chronic times since injury, our descriptive findings do not enable conclusions about within group changes in FC over time. Nevertheless, cross-sectionally, patients with TBI who were imaged in the acute and acute/subacute window showed less increased (42%) than decreased connectivity (58%) as compared to controls (Figure 5). From the subacute to chronic window however, the differences flipped to more increased (60%) than decreased connectivity (40%) as compared to controls. Though these results are cross-sectional, this trend suggests connectivity may increase over time post-injury.

Several of the studies included in our review contained longitudinal data, the majority of which report a relative within-group increase in connectivity at the chronic as compared to the more acute time points post-injury (21, 42, 43). Studies that did not find increased connectivity in the chronic time points showed no difference as a function of time since injury (44, 45) or revealed more complex findings, such as increased and decreased connectivity within a single network as a function of time since injury (e.g., increased connectivity at the left anterior prefrontal cortex, but decreased connectivity at the middle cingulate gyrus) (46). Most notably, however, no longitudinal study reported a whole-network decrease in connectivity in the chronic stage. Studies utilizing graph theory have also noted a pattern of initially decreased to subsequently increased connectivity over time since injury (15, 47, 48). Increased connectivity has correlated with better function (42) and worse function (49) demonstrating again that the direction of connectivity change or difference compared to control may not be as important as the finding of any change or difference. These longitudinal effects have been interpreted as adaptive or compensatory when relating to improved function (44, 50, 51).

### Age

Parsing the comparisons by age did not reveal any discernible pattern. Trends based on age alone (adolescent vs. adult) are difficult to draw given the large majority (90%) of studies included only adults. Furthermore, adult age ranges were restricted, with the first quartile less than 26.88 years and the fourth quartile greater than 38.49 years. Because most of the TBI patients were scanned in early adulthood, we cannot infer whether connectivity patterns emerge after injury in younger and older ages due to the paucity of studies overall, and in our study in particular, for these age groups.

### Population type

Parsing comparisons by population type revealed a notably larger percent signal (71%) in the military population. Overall, however, the military population showed an even split of increased and decreased connectivity findings, which is consistent with the other populations studied. The military population was also the least studied (7.8% of total comparisons). Because injury in military personnel can include unique mechanisms and are often comorbid with sequelae of psychological trauma as compared to civilian and sport-related injuries (52), it is possible that the brain differences are more striking.

### Networks

The DMN was the most studied network (78 comparisons) and had the highest signal of any of the networks included (55% signal). The ratio of increased to decreased connectivity between people with TBI and matched controls was, however, roughly equal (23–20, respectively). It is well known that the DMN plays a critical role in intrinsic cognitive processes. Though the DMN generally had mixed results across most patient characteristics, it trended toward increased connectivity in the moderate/severe TBI population and at the subacute time frame (Figure 6b, column 1). Previous studies have interpreted this increased connectivity as a compensatory or adaptive mechanism because of its association with positive cognitive outcomes (44, 50, 51). It is possible compensatory processes do not readily occur unless the injury is sufficiently damaging. Perhaps such compensation does not occur until at least weeks to months after the injury, which may follow periods of increased metabolic activity (53, 54).

The DAN showed the lowest signal (14%) of all the networks, suggesting this network may not be affected by TBI and/or whatever changes that may occur are not sensitive to rsfMRI methods. Of note, the DAN also had the lowest number of total comparisons across the literature (Figure 4). Further investigation of the DAN may be required to rule out the DAN as a potential marker of TBI. Alternatively, this may be a network to leave out of analyses to retain valuable degrees of freedom.

The SN had the next lowest percent signal (39%) (Figure 4). Given the putative role of the SN in affective processes (55, 56), including affective dysregulation in some patients with TBI (57), its low signal is surprising. However, because the SN serves as a switch between intrinsic and extrinsic cognitive processes (58), rsfMRI may not be sensitive enough to capture changes in connectivity without patients performing goal-directed activity or without looking at internetwork connectivity differences. Again, further investigation of this network at rest and during tasks will provide greater insight to its sensitivity as a potential biomarker of TBI.

### Methodological factors

Parsing the comparisons by whether patients were instructed to keep their eyes open, fixated, or closed revealed a striking pattern. Eyes open or fixated showed 75 and 64% signal, respectively, whereas eyes closed only revealed 33% signal (Figure 7). Interestingly, about half of the studies instructed participants to close their eyes, despite newer evidence that eyes open may enhance connectivity strengths (59).

Generating quartiles of the number of volumes captured during scans revealed a notably higher percent signal among the third quartile (73%), which had a range of 182–245 volumes per scan (Figure 7). In contrast, all other quartiles had a markedly lower percent signal (<48%). These results suggest the number of volumes captured during scans may have an ideal value that could serve to increase the sensitivity of rsfMRI in detecting connectivity differences.

Generating quartiles of the scan duration revealed a notably lower percent signal among the second quartile (37%), which had a range of 315–360 s (Figure 7). In contrast, the first, third, and fourth quartiles all demonstrated markedly higher percent signals. Recent studies support greater reliability with increasing scan length, though that cutoff point is debated and not well defined (60, 61).

Other than GE, which was rarely used, scanner manufacturer and coil channel did not meaningfully impact the findings in that there were similar proportions of increased, decreased, and null findings no matter which scanner or head coil was used (Figure 7). GE did demonstrate a percent signal of 77% suggesting more likely to show some difference across groups as compared to null but only four studies utilized GE scanners, which is not enough to make a strong conclusion. However, the fact that the other two most commonly used scanner manufacturers did not appreciably impact the findings suggests that this pattern of findings are likely generalizable and could be expected to hold across sites in future research.

Creating tertiles of the slice thickness demonstrated a substantially higher percent signal among the third tertile, which had a thickness of >4 mm per slice (Figure 7). The choice in slice thickness is determined by the time to acquire a set brain volume, spatial resolution, minimization of signal dropout, and BOLD signal-to-noise (SNR) ratio, which largely determines the statistical power to detect nodal activation. Therefore, any increase in spatial resolution with thinner slices will be offset by a reduction in the statistical significance due to increased SNR (62–64). Prior work demonstrates that thicker slices will yield more low-percentage signal changes, consistent with our results (63). However, the optimal slice thickness depends on the study, as certain brain regions may require higher resolution, which may come at the cost of sensitivity per unit time, to enhance localization and reduce signal dropout.

When evaluating preprocessing software as a factor, custom pipelines achieved a notably higher signal than the other softwares (Figure 8). It may be that custom pipelines are designed for the specific dataset obtained, and thus software can be designed to achieve higher sensitivity in detecting connectivity differences. However, because this study did not have access to the custom softwares, there is the possibility that a higher signal could result from poorly implemented pipelines that are not as rigorous at mitigating specious findings.

Parsing the comparisons by regression type revealed performing regression in all categories (global, white matter, CSF), except motion, increased the likelihood of detecting a significant difference between TBI and control populations (Figure 8). Because percent signal was similar between comparisons that did and did not perform motion regression, it appears that motion regression does not significantly affect sensitivity of finding a difference in connectivity in these studies.

## Limitations

It is impossible to account for all the factors that contribute to the heterogeneity in neuroimaging of TBI. This study aimed to isolate individual variables across 50 studies, but it is important to acknowledge that many of these variables may influence one another (e.g., most moderate/severe TBI cases were studied in the chronic phase). MRI sequence parameters vary between sites, evolve over time, and may interact in ways that impact results. Inconsistent reporting of certain parameters (e.g., acquisition direction, reconstruction plane, slice thickness), made it difficult to extract and parse our findings by an exhaustive list of sequence parameters. Nevertheless, we extracted the parameters we found to be most consistently reported and that previous work suggests may impact results. Therefore, careful consideration should be taken when interpreting individual variables as interactions may confound results.

Given discrepancies in the number of comparisons within categories (e.g., majority of comparisons in mild TBI, middle age range, civilian population, default mode network), we caution making strong conclusions for variables that received less attention (e.g., much less comparisons in more severe injuries, younger and older ages, dorsal attention network). This is a limitation of our study, but also reflective of the current assumptions for what might be most biologically or clinically important in this field.

Given the large quantity of results generated in rsfMRI research, there exists a severe underreporting of null findings throughout the literature. However, reported results would be unreadable if the entirety of results were explicitly reported. We chose not to infer null findings when primary authors did not clearly report null findings as interpretation would be prone to inconsistency. This aspect of rsfMRI literature makes it difficult to gather an accurate representation of percent signals across the literature, and thus our percent signals are likely skewed toward higher values.

## Recommendations for future studies

As discussed throughout this review, there are multiple factors that may contribute to the heterogeneity of rsfMRI connectivity patterns in TBI. Parsing the findings by these factors, however, did not reveal a dramatic consensus, which strongly supports the need for consistency and best practices across studies. Below, we make several recommendations to establish greater consistency and reproducibility for future studies. We also identify areas of study where literature is lacking and therefore encourage greater investigation.

1. **Networks:** The low percent signal observed in the DAN and SN suggest they may not be affected by TBI and/or are not sensitive to rsfMRI methods. We recommend that future studies pay particular attention to the DAN and SN to provide greater insight into their sensitivity. However, their low sensitivity would make it reasonable to exclude these networks from analysis to preserve degrees of freedom.
2. **Demographics:** Functional connectivity in TBI may be affected by multiple demographic factors, such as severity, chronicity, age, and population or mechanism of TBI. We encourage future studies to either narrow their demographic inclusion criteria to evaluate subgroups within heterogeneous populations or obtain large enough sample sizes to account for the heterogeneity. Some of these challenges are seemingly insurmountable. However, in the event that sample size is limited or heterogeneous, combining datasets across sites or with freely downloadable datasets might provide the power needed for more reliable analyses through techniques such as harmonization and meta-analysis. ENIGMA (65) and FITBIR (66) are both viable solutions for this challenge.
3. **Age:** Age range was restricted to early-middle adulthood across the studies, and thus the current literature has yet to evaluate changes in connectivity across child and elderly populations. We encourage future studies to focus on these populations that may be potentially more vulnerable.
4. **Injury mechanism:** Restricting studies to a specific injury mechanism, rather than population type, may help to reduce variability. For example, studies could focus on car accidents, blast exposure, or sport-related injuries. Thus, studies should narrow their inclusion criteria to obtain a more focused assessment of mechanisms’ role in functional alterations.
5. **Longitudinality:** The vast majority of studies are cross-sectional rather than longitudinal. Adding multiple imaging time points to study designs will effectively increase power, make it possible to derive changes over time, and reduce natural variability in rsfMRI patterns.
6. **Canonical network:** We recommend future studies classify results to an established canonical network to facilitate interpretation. It is critical for replication that authors clearly indicate how regions of interest or networks were derived and what expected differences they hypothesize.
7. **Methodology:** We recommend future studies utilize fixated or open eye scans as well as volume ranges between 182 and 245 volumes given their relatively higher sensitivity in signal detection. Though scan duration had mixed findings, percent signal was consistently higher among longer scan durations. In line with previous literature, we also recommend utilizing scan durations of at least 360 s.
8. **Regression:** All forms of regression, except motion regression, yielded higher sensitivity than studies that did not perform regression. In any case, we recommend all studies clearly identify whether they performed global, CSF, white matter, and motion regression as this ensures future studies can either replicate or refute the findings in independent samples.
9. **Preprocessing software:** Though recommendations on specific software is beyond the scope of this paper, FSL and SPM8 had the highest sensitivity in detecting a signal. Studies utilizing custom software are encouraged to also process their data with established software to compare and confirm results.

## Conclusion

Despite the obvious potential for neuroimaging as a biomarker in TBI, there seems to be no clear set of rsfMRI findings that universally differentiate the injured from healthy brain across all injury severities, mechanisms, chronicities, and population types. Although it is reasonable to hypothesize the heterogeneity in TBI populations studied drives the heterogeneity of findings, it is unclear from our study that any one factor (e.g., age, chronicity, population type, etc.) was a strong predictor of connectivity trends. Our study confirms that rsfMRI literature is largely mixed when considering connectivity differences between brain injured and healthy individuals no matter how the data is parsed. We identified a few patterns that may reflect mechanisms of brain injury or recovery, but ultimately our findings highlight the need for multi-site and longitudinal studies, greater methodological consensus across studies, and/or consortia to combine data retrospectively using the same analysis methods. These findings will guide future research and should help clinicians understand the early state of advanced imaging modalities, like rsfMRI, which are not yet ready for clinical use in individual patients.

## Data Availability

The original contributions presented in the study are included in the article/supplementary material, further inquiries can be directed to the corresponding author.

## Footnotes

^1^https://github.com/danielfrees/rsfMRI_LitReview
